# Decreased Bone Mineral Density in Adults Born with Very Low Birth Weight: A Cohort Study

**DOI:** 10.1371/journal.pmed.1000135

**Published:** 2009-08-25

**Authors:** Petteri Hovi, Sture Andersson, Anna-Liisa Järvenpää, Johan G. Eriksson, Sonja Strang-Karlsson, Eero Kajantie, Outi Mäkitie

**Affiliations:** 1 Hospital for Children and Adolescents, Institute of Clinical Medicine, University of Helsinki, Helsinki, Finland; 2 Department of Chronic Disease Prevention, National Institute for Health and Welfare, Helsinki, Finland; 3 Department of General Practice and Primary Health Care, Institute of Clinical Medicine, University of Helsinki, Helsinki, Finland; 4 Vasa Central Hospital, Vasa, Finland; Leiden University Medical Centre, Netherlands

## Abstract

**Background:**

Very-low-birth-weight (VLBW, <1,500 g) infants have compromised bone mass accrual during childhood, but it is unclear whether this results in subnormal peak bone mass and increased risk of impaired skeletal health in adulthood. We hypothesized that VLBW is associated with reduced bone mineral density (BMD) in adulthood.

**Methods and Findings:**

The Helsinki Study of Very Low Birth Weight Adults is a multidisciplinary cohort study representative of all VLBW births within the larger Helsinki area from 1978 to 1985. This study evaluated skeletal health in 144 such participants (all born preterm, mean gestational age 29.3 wk, birth weight 1,127 g, birth weight Z score 1.3), and in 139 comparison participants born at term, matched for sex, age, and birth hospital. BMD was measured by dual energy X-ray absorptiometry at age 18.5 to 27.1 y. Adults born with VLBW had, in comparison to participants born at term, a 0.51-unit (95% confidence interval [CI] 0.28–0.75) lower lumbar spine Z score and a 0.56-unit (95% CI 0.34–0.78) lower femoral neck Z score for areal BMD. These differences remained statistically significant after adjustment for the VLBW adults' shorter height and lower self-reported exercise intensity.

**Conclusions:**

Young adults born with VLBW, when studied close to the age of peak bone mass, have significantly lower BMD than do their term-born peers. This suggests that compromised childhood bone mass accrual in preterm VLBW children translates into increased risk for osteoporosis in adulthood, warranting vigilance in osteoporosis prevention.

*Please see later in the article for the Editors' Summary*

## Abstract 2

Petteri Hovi and colleagues evaluate skeletal health in 144 adults born preterm with very low birth weight and show that as adults these individuals have significantly lower bone mineral density than do their term-born peers.

## Editors' Summary

**Background:**

Most pregnancies last 40 weeks but some babies arrive earlier than expected. Sadly, babies born before 37 weeks of pregnancy—premature babies—are more likely to die than full-term babies, although recent improvements in neonatal care have increased their chances of survival. Premature babies also often have serious long-term health problems, particularly those born before 32 weeks of pregnancy. Such extremely premature babies have poorly developed internal organs and are usually very small—babies whose birth weight is less than 1,500 g are called very-low-birth-weight (VLBW) babies; the average full-term birth weight is about 3,500 g. Furthermore, their bones are not as well developed as those of full-term babies. The human skeleton initially consists of a soft fibrous material called cartilage. This is gradually transformed into bone by a process called bone mineralization. The last third of pregnancy is a crucial period for bone mineralization although the process continues throughout infancy and childhood. Thus, VLBW babies often have subnormal skeletal mineralization and their accrual of bone mass during childhood is frequently compromised.

**Why Was This Study Done?:**

It is not known whether the childhood bone deficits of VLBW babies persist into adulthood because the first generation of these infants not to die soon after birth is only just reaching adulthood. Peak bone mass is reached in early adulthood (bone mass begins to decrease from the age of 35 years onward) and is an important indicator of whether an individual will develop osteoporosis (thinning of the bones) and be susceptible to bone fractures later in life. If adults with VLBW (about 1% of live births in high-income countries are now VLBW births) do have a subnormal peak bone mass and reduced bone mineral density (BMD), they may be able reduce their risk of developing osteoporosis by eating a healthy diet and exercising regularly. In this study (part of the Helsinki Study of Very Low Birth Weight Adults), the researchers investigate the skeletal health of people who were born with VLBW in the Helsinki area between 1978 and 1985.

**What Did the Researchers Do and Find?:**

The researchers compared the skeletal health of 144 young adults who were born prematurely with VLBW and subnormal BMD with that of 139 age- and sex-matched individuals who were born at term. They measured the BMD of the participants (average age 22.6 years) using “dual energy X-ray absorptiometry” and determined a “Z score” for the spine in the lower back (the lower lumbar spine) and the hip (two sites that are routinely examined in assessments of skeletal health). Z scores indicate whether an individual's BMD is significantly different from the average BMD of healthy age- and sex-matched people; in this study, reduced BMD was defined as a Z score of −1.0 or less. The researchers found that adults born with VLBW had an average Z score of −0.51 at the lower lumbar spine and −0.56 at the hip when compared with the adults born at term. Furthermore, 44% of the VLBW participants but only 26% of the term-born participants had a lumbar spine Z score of −1.0 or less. Adjustment for the shorter height of the VLBW participants slightly reduced these differences in BMD but the differences remained statistically significant.

**What Do These Findings Mean?:**

These findings show that, when studied close to the age of peak bone mass, young adults born with VLBW have a significantly lower BMD than their term-born peers and a 2-fold greater risk of having a lumbar spine Z score of below −1.0; a unit decrease in Z score approximately doubles the risk of bone fractures. Because BMD measurements were only taken at one age, it remains possible, however, that the BMD of the VLBW adults might eventually match that of their full-term peers. Recently born VLBW babies still have a lower than average BMD during their childhood, note the researchers, even though their care has changed since the people included in this study were born. Thus, these findings suggest that people who were VLBW infants should be encouraged to eat food rich in vitamin D and calcium and to do regular weight-bearing exercise throughout their lives to improve their bone health and reduce their risk of developing osteoporosis.

**Additional Information:**

Please access these Web sites via the online version of this summary at http://dx.doi.org/10.1371/journal.pmed.1000135.

- The March of Dimes, a nonprofit organization for pregnancy and baby health, provides information on preterm birth (in English and Spanish)
- The Nemours Foundation, another nonprofit organization for child health, also provides information on premature babies (in English and Spanish)
- MedlinePlus provides links to other information on premature babies and to information on osteoporosis (in English and Spanish)
- The US National Institute of Arthritis and Musculoskeletal and Skin Diseases and the UK National Health Service also provide detailed information on all aspects of osteoporosis
- Further details about the Helsinki Study of Very Low Birth Weight Adults are available

## Introduction

The last trimester of pregnancy is crucial for fetal bone mineralization. Fetal and maternal mineral metabolism is uniquely adapted to provide sufficient calcium and other minerals to fully mineralize the fetal skeleton before birth [1]. The interplay of minerals and calciotropic hormones is important, but for appropriate intra-uterine skeletal development, active fetal movement against the uterine wall is needed as well. Mineralization occurs rapidly in late gestation; up to 80% of the body calcium of a term newborn accrues during the third trimester [2].

Individuals born significantly preterm are deprived of the positive skeletal effects of the last trimester. Preterm birth (gestational age <37 wk) and low birth weight may result from abnormal pregnancy, involving, for example, preeclampsia or infection, which may influence skeletal development. Regardless of the reason for preterm birth, preterm infants are postnatally exposed to several factors that may further adversely affect bone health, including medications, immobilization, compromised respiration, nutritional problems, and infections. Not surprisingly, preterm birth results in subnormal skeletal mineralization and compromised bone-mass development in the neonatal period and early childhood [3]–[5].

Infants with very low birth weight (VLBW, <1,500 g) represent the extreme lower end of the prematurity spectrum. Their survival has significantly improved during recent decades [6],[7], and the first generation who received modern neonatal care is now entering adulthood. At present, among live births in high-income countries, VLBW infants constitute approximately 0.9% to 1.5% [8],[9]. In childhood they have subnormal bone mass as compared with their term-born peers [4],[10],[11]. Whether recovery takes place before attainment of peak bone mass is unknown. We assessed bone mineral density (BMD) in young adults within the Helsinki Study of Very Low Birth Weight Adults [12].

## Methods

### Ethics Statement

The study complies with the guidelines of the Declaration of Helsinki. The study protocol was approved by the ethics committee at the Helsinki University Central Hospital, and all participants gave their written informed consent.

### Selection of VLBW and Term-Born Individuals

Of a total of 474 consecutive VLBW infants born between January 1978 and December 1985, 335 were discharged alive from the neonatal intensive care unit of Children's Hospital at Helsinki University Central Hospital, the only tertiary neonatal care centre in the province of Uusimaa, Finland, population 1.1 million [12],[13]. In 2004, a comparison group was selected from the records of all consecutive births: For each VLBW survivor, the next term-born singleton infant was selected as a comparison person. This newborn infant had to be of the same sex and be born in the same birth hospital with a birth weight not small for gestational age (not SGA, >−2.0 standard deviation [SD]).

### Perinatal Data, Recruitment in Young Adulthood

**Figure 1:**
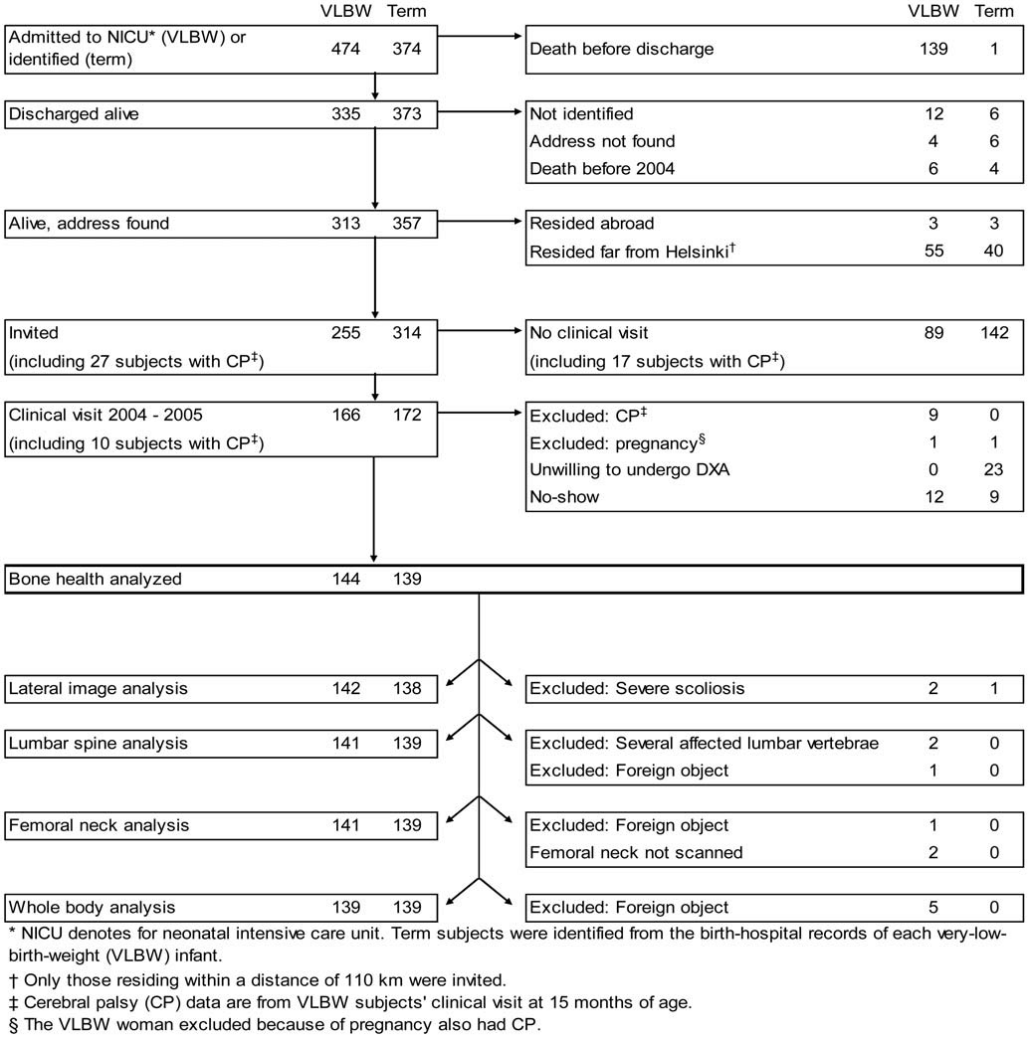
Flow chart.

The individuals in each cohort were traced through the Population Register Centre in 2004 as young adults. For the VLBW individuals, mortality from hospital discharge to June 2004 was 1.8% and for the individuals born at term, 1.1%. Of the survivors, 95.1% and 96.7% were traced (Figure 1). Those 255 VLBW and 314 term-born individuals residing in the greater Helsinki area were invited to participate in a clinical study assessing several indicators of their adult health, including BMD. Two pregnant women, one per group, were not scanned; nine VLBW individuals with cerebral palsy were excluded from analysis since severe immobility may affect skeletal health [14]. Bone densitometry required a separate visit and 12 VLBW and 32 term-born individuals did not undergo it. As a result, of the invited persons without cerebral palsy and without known pregnancy, 144 (63.4%) with VLBW and 139 (44.4%) who were term-born were analyzed for BMD (Figure 1). Data on intensive care were stored into research files during the neonatal-intensive-care-unit stay. Maternal welfare clinic and hospital records provided perinatal and neonatal data [12]. Maternal preeclampsia was defined as proteinuria and blood pressure >140/90 after postmenstrual week 20 [15]. Rupture of membranes was defined as premature, if occurring more than 24 h prior to birth, and bronchopulmonary dysplasia [16] as oxygen demand at age 28 d. Weights at birth and at 36 and 40 wk of postmenstrual age were assessed after conversion to SD scores [17]. Everybody involved in data collection was blinded for the participants' birth weight and other characteristics.

### Clinical Data

At study assessment, at mean age 22.6 y (SD 2.2), range from 18.5 to 27.1 y, all participants were measured for height and weight. They recorded the brand names and amounts of all food items and nutritional supplements consumed during 2 working days and 1 weekend day. Average daily intake of calcium and vitamin D was then calculated for each individual based on the Finnish Food Composition Database [18]. Leisure-time physical activity was assessed with questions on exercise intensity (four categories), exercise frequency (six), and exercise duration (four). Participants reported their parents' body heights; they were converted into a target height SD score, which is a standard measure of genetic growth potential in pediatric practice [19]. Participants reported their current medications and their parents' present education, which was then categorized into three levels, with the higher-educated parent's level serving as an indicator of family socioeconomic status.

### Bone Densitometry

Bone mineral content (BMC) and areal BMD for the lumbar spine (L1–L4), femoral neck, and whole body, as well as body composition, were measured with dual-energy X-ray absorptiometry (DXA, Hologic Discovery A, software version 12.3∶3) and transformed into Z scores on the basis of the absorptiometry equipment-, age-, and sex-specific reference database. T scores characterize BMD in comparison to that of healthy young adults, and the WHO defines postmenopausal osteoporosis as a T score ≤−2.5 and osteopenia as a T score >−2.5 and ≤−1.0 [20]. For the present study, however, we used Z scores to adjust for age, and we chose a cut-off Z score value of −1.0 to define reduced BMD. To minimize the effect of bone size on lumbar spine BMD, an estimate [21] of volumetric density was also calculated: (bone mineral apparent density [BMAD] = BMC_L1–4_/bone area_L1–4_^1.5^).

An experienced reader (OM), blinded to the participants' identity, birth weight, and other characteristics, evaluated lateral images of the thoracic and lumbar spine from T4 to L4 for identification of possible vertebral compression deformities. The vertebrae were compared with adjacent vertebrae and graded depending on percent reduction in anterior, mid-, or posterior vertebral height: normal, below 20%; grade 1, 20%–25%; grade 2, 25%–40%; and grade 3 deformity, ≥40% [22]. The DXA scanner-derived images could not be evaluated in two VLBW and one term-born individual because of severe scoliosis. Scans with foreign objects (surgical fixation material, jewelry) in the measurement area (five people) were omitted from analysis. If more than one lumbar vertebra was compressed, the corresponding lumbar spine scan was excluded from analysis (two people); if only one lumbar vertebra was compressed, BMD without the affected vertebra was used (seven people).

### Statistical Analysis

All analyses were performed with SPSS for Windows, Version 15.0. All significance tests were two-sided with a type I error set at 0.05. It was estimated that an existing difference of 0.40 in the SD score can be detected with a statistical power of 90% by assessing 140 participants per group. In a secondary analysis requiring food-diary data, which was available for 112 VLBW and 86 term-born participants, the corresponding power was 79%. Pearson's chi-squared test served to compare proportions, Student's *t*-test to compare means; in case of a strongly right-skewed distribution (duration of mechanical ventilation), the Mann-Whitney U-test was utilized to compare medians, and Spearman's correlation to study associations. Other right-skewed distributions attained normality after logarithmic transformation; the group-mean differences were estimated and tested with linear regression analysis, odds ratios with logistic regression. Age was introduced as a confounder to models predicting BMAD and BMC; since bone mass increases until it reaches a plateau at age 20 y, a piecewise linear function allowed a different effect for increasing age before and after age 20 y [23],[24]. Height, weight, and body mass index (BMI) have been shown to associate with bone mass [3],[25], and height and BMI were forced into the models. BMI was chosen because it is a measure of body weight with minimal correlation to height. Typical exercise intensity, frequency, or duration, as well as socioeconomic status and the intake of vitamin D and calcium were considered as potential confounders but were omitted from the final models if this did not change the effect of VLBW. Since all outcome differences between the VLBW and term-born groups were similar regardless of sex (*p*-values for interaction >0.10), the main findings, adjusted for sex, are represented with men and women combined. When analyzing any VLBW group it is notable that a weight cut-off causes an association of a smaller birth weight SD score and a higher gestational age. In our VLBW group, of those born after completing 32 full wk of gestation, 22 out of 26 individuals were SGA. In addition, of those born before completing 28 full wk only one out of 31 individuals was SGA. In order to reduce any effects of this artificial association, all VLBW participants who were born SGA were excluded from a secondary analysis assessing effects of gestational age on the outcomes. Likewise, those outside gestational age range of 28 to 31 full wk were excluded from another secondary analysis assessing effects of SGA birth.

## Results

### Cohort Characteristics

In the two groups, birth weights ranged from 600 to 1,500 g and from 2,560 to 4,930 g, and gestational ages from 24.0 wk to 35.6 wk and from 37.0 to 42.9 wk (Table 1). Of the VLBW participants, 21 (14.6%) were from a twin and five (3.5%) from a triplet pregnancy. Eligible participants included in BMD analysis or without BMD measurement were similar, in terms of maternal chorionamnionitis or premature rupture of the membranes (Pearson's chi-squared test), placental weight, birth-weight Z score, and gestational age (Student's *t*-test); and duration of mechanical ventilation (Mann-Whitney U-test).

**Table 1:** Characteristics of VLBW infants compared to those of term born infants.

Characteristics	VLBW	Term
**Participants, ** ***n***	144	139
**Males, ** ***n*** ** (%)**	60 (41.7)	55 (39.6)
**Maternal preeclampsia** a **, ** ***n*** ** (%)**	32 (22.2)	11 (7.9)
**Maternal hypertension in pregnancy without proteinuria** a **, ** ***n*** ** (%)**	5 (3.5)	21 (15.1)
**Maternal smoking during pregnancy** a **, ** ***n*** ** (%)**	28 (19.4)	20 (14.4)
**Placental weight, g, mean (SD)**	435 (186)	649 (125)
**Birth weight, g, mean (SD)**	1,127 (218)	3,600 (471)
**Gestational age, wk, mean (SD)**	29.3 (2.2)	40.1 (1.1)
**SD score for birth weight, mean (SD)**	−1.3 (1.5)	0.1 (1.0)
**SD score for birth weight <−2.0, ** ***n*** ** (%)**	49 (34.0)	0 (0)
**Maternal chorionamnionitis** b **, ** ***n*** ** (%)**	26 (18.1)	
**Premature rupture of the membranes** b **, ** ***n*** ** (%)**	24 (16.7)	
**Culture-positive sepsis during neonatal period** b **, ** ***n*** ** (%)**	11 (7.6)	
**Mechanical ventilation** b **, d, median (25th and 75th percentile)**	5 (0–14)	
**Oxygen** b **, d, median (25th and 75th percentile)**	13 (3–33)	
**Bronchopulmonary dysplasia** b **, ** ***n*** ** (%)**	26 (18.1)	

a *p*-Value was 0.001 for VLBW versus term babies for maternal preeclampsia, 0.001 for maternal hypertension, and 0.26 for maternal smoking (Pearson's chi-squared test).

b For the VLBW group only, data were collected during neonatal intensive care.

Compared with participants born at full term, in young adulthood VLBW men were 5.1 cm (95% confidence interval [CI] 2.6–7.6, *p<*0.001) shorter and women 4.8 cm (95% CI 2.7–6.9, *p<*0.001) shorter, the geometric mean of VLBW individuals' BMI was 1.5 kg/m^2^ (95% CI 0.3–2.6, *p = *0.02) lower among men and 0.8 kg/m^2^ (95% CI −0.3 to 1.8, *p = *0.14) lower among women. Median daily calcium intake for the VLBW and term-born groups was, respectively, 831 mg (*n* = 112, interquartile range [IQR] 555–1,120 mg) and 953 mg (*n* = 86, IQR 715–1,240 mg, *p = *0.003, Student's *t*-test after logarithmic transformation) and median daily vitamin D intake 3.1 µg (IQR 1.9–4.4 µg) and 3.3 µg (IQR 2.1–6.0 µg, *p = *0.13, Student's *t*-test after logarithmic transformation). Frequency of leisure-time physical exercise in the two groups was similar, but typical exercise duration and intensity were lower for adults with VLBW (Table 2).

**Table 2:** Clinical characteristics at study assessment in young adulthood.

Characteristic	Category	VLBW	Term	*p*-Value
**Men/women, ** ***n***		60/84	55/84	0.72
**Age, y**		22.6 (2.2)	22.6 (2.2)	0.89
**Height, cm**	Men	175.4 (7.2)	180.5 (6.2)	<0.001
	Women	162.2 (7.5)	167.0 (6.5)	<0.001
**Height SDS** a	Men	−0.45 (1.06)	0.30 (0.92)	<0.001
	Women	−0.49 (1.31)	0.35 (1.14)	<0.001
**Target height SDS** b	Men	0.46 (0.64)	0.43 (0.62)	0.79
	Women	0.37 (0.64)	0.43 (0.63)	0.54
**Weight** c **, kg**	Men	67.5 (1.18)	76.3 (1.14)	<0.001
	Women	57.3 (1.20)	63.0 (1.18)	<0.001
**BMI** c **, kg/m^2^**	Men	22.0 (1.18)	23.4 (1.13)	0.02
	Women	21.8 (1.17)	22.6 (1.17)	0.14
**Leisure time exercise frequency**	None	6 (4.3)	4 (2.9)	0.42
	Less than once per month	19 (13.5)	16 (11.5)	
	Once or twice a month	19 (13.5)	10 (7.2)	
	Once a week	30 (21.3)	31 (22.3)	
	2 to 3 times a week	38 (27.0)	52 (37.4)	
	4 to 5 times a week	15 (10.6)	15 (10.8)	
	Daily	14 (9.9)	11 (7.9)	
**Leisure time exercise typical duration**	<30 min	21 (15.1)	6 (4.4)	<0.001
	30–60 min	54 (38.8)	35 (25.5)	
	1–2 h	60 (43.2)	83 (60.6)	
	≥2 h	4 (2.9)	13 (9.5)	
**Leisure time exercise intensity**	Walking	42 (30.4)	16 (11.7)	<0.001
	Walking or light running	39 (28.3)	34 (24.8)	
	Light running	38 (27.5)	40 (29.2)	
	Brisk running	19 (13.8)	47 (34.3)	
**Calcium intake** c **, mg/day**		761 (1.81)	958 (1.58)	0.003
**Phosphate intake** c **, mg/day**		1195 (1.48)	1386 (1.39)	0.005
**Vitamin D intake** c **, µg/day**		3.0 (1.92)	3.4 (1.93)	0.13

a Height SDS by Finnish growth charts.

b Target height SDS reflects the expected adult height of the individual based on genetic growth potential. It was calculated from the equation: 0.0611×father's height (cm)+0.0703×mother's height (cm) − 22.1 [19].

c Numbers represent geometric means (and geometric SDs) for the intakes, weight, and BMI; and means (and SD) or *n* (%) for the other variables. Geometric SD corresponds to relative increase in the variable corresponding to 1 SD-unit absolute increase in the logarithm of the variable. Number of participants not providing valid data were 13 on both parents' heights, eight on exercise habits, and 86 on food-diary data. *p-V*alues are for the difference between the VLBW and term-born groups (Student's *t*-test or Pearson's chi-squared test).

### Skeletal Health

We first assessed compression deformities in thoracic or lumbar vertebrae from lateral images that could be evaluated in 142 VLBW and 138 term-born individuals: 17 with VLBW and 19 of the term-born had a significant compression deformity in at least one thoracic or lumbar vertebra (*p = *0.65). We found multiple compression deformities in ten VLBW and seven term-born participants (*p = *0.49); two VLBW participants had multiple compression deformities in the lumbar spine. A term-born man had two vertebrae classified as grade 2; the others had grade 1 vertebral deformities.

**Figure 2:**
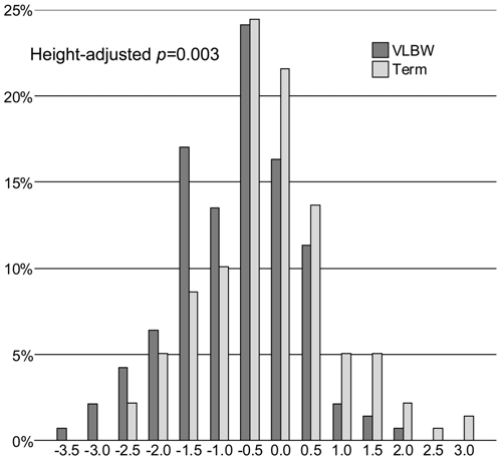
Lumbar spine BMD distributions in VLBW and term-born young adults. Mean lumbar spine BMD Z score, as provided by the absorptiometry equipment, was lower in the 141 young adults with VLBW (<1,500 g), dark bars, than in the 139 term-born comparison participants (*p*<0.001). Each category includes and is denoted by its' highest value.

As the primary outcome, we compared BMD in young adults with VLBW with that in individuals born at term. BMD Z scores in VLBW individuals were 0.51 (95% CI 0.28–0.75) units lower in the lumbar spine, 0.56 (0.34–0.78) units lower in the femoral neck, and 0.33 (0.11–0.56) units lower in the whole body (Figure 2; Tables 3 and 4). In categorized analysis, 44% of the VLBW individuals and 26% of the term-born individuals had a lumbar spine Z score ≤−1.0; this corresponds to VLBW individuals having 2.3-fold odds (95% CI 1.4–3.8) of having a lumbar spine Z score ≤−1.0. After adjusting for VLBW individuals' shorter height, the odds ratio was 1.8 (95% CI 1.0–3.1). Adjustment for the VLBW adults' shorter height and lower BMI attenuated the differences in BMD. However, all differences in lumbar spine and femoral neck BMD remained statistically significant. Also the lumbar spine BMAD, which takes the VLBW individuals' smaller bone size into account, was lower in VLBW individuals (Tables 3 and 4).

**Table 3:** Bone health at study assessment in young adulthood.

Characteristic	Sex	VLBW	Term	*p*-Value
**Number of men/women**	—	60/84	55/84	0.72
**Whole body fat, percent of body weight**	Men	18.0 (6.4)	18.4 (5.7)	0.72
	Women	29.6 (6.1)	30.0 (5.5)	0.67
**Whole body lean mass, kg**	Men	54.4 (8.1)	61.6 (7.9)	<0.001
	Women	39.2 (5.6)	43.0 (5.6)	<0.001
**Whole body lean mass adjusted for height, kg**	Men	56.5 (6.4)	59.3 (6.7)	0.02
	Women	40.4 (3.8)	41.5 (4.7)	0.10
**Lumbar spine aBMD Z score**	—	−0.93 (0.98)	−0.41 (1.05)	<0.001
**Lumbar spine BMAD**	—	0.123 (0.013)	0.127 (0.014)	0.007
**Femoral neck aBMD Z score**	—	−0.42 (0.85)	0.14 (0.97)	<0.001
**Whole body aBMD Z score**	—	−0.59 (0.87)	−0.26 (1.02)	0.003
**Whole body BMC, g**	Men	2,375 (389)	2,677 (410)	<0.001
	Women	1,949 (263)	2,122 (258)	<0.001
**Whole body bone area, cm^2^**	Men	2,140 (215)	2,321 (167)	<0.001
	Women	1,855 (195)	1,985 (158)	<0.001
**Whole body less head BMC** a **, g**	Men	1,873 (346)	2,169 (369)	<0.001
	Women	1,463 (227)	1,623 (230)	<0.001
**Whole body less head bone area** a **, cm^2^**	Men	1,901 (207)	2,067 (162)	<0.001
	Women	1,637 (190)	1,756 (153)	<0.001

Numbers represent means (and SD). *p-V*alues are for the difference between the VLBW and term-born groups (Student's *t*-test or Pearson's chi-squared test). aBMD denotes areal bone mineral density. SGA, birth weight SD score ≤−2.0.

a Whole body less head BMC and bone area contain the whole body with head excluded.

**Table 4:** Difference between VLBW and the term born young adults: multiple linear regression models.

Outcome	*n*	Model^a^	Difference (95% CI)^b^	*p-*Value
**Lumbar spine aBMD Z score**	280	1	−0.51 (−0.75 to −0.28)	<0.001
	280	2	−0.37 (−0.62 to −0.13)	0.003
	280	3	−0.25 (−0.48 to −0.02)	0.03
	272	4	−0.26 (−0.51 to −0.01)	0.04
**Lumbar spine BMAD**	280	1	−0.0043 (−0.0075 to −0.0012)	0.006
	280	2	−0.0057 (−0.0090 to −0.0024)	<0.001
	280	3	−0.0040 (−0.0071 to −0.0010)	0.01
	272	4	−0.0041 (−0.0074 to −0.0007)	0.02
**Femoral neck aBMD Z score**	280	1	−0.56 (−0.78 to −0.34)	<0.001
	280	2	−0.48 (−0.71 to −0.26)	<0.001
	280	3	−0.35 (−0.55 to −0.14)	<0.001
	272	4	−0.40 (−0.64 to −0.17)	<0.001
**Whole body aBMD Z score**	278	1	−0.33 (−0.56 to −0.11)	0.003
	278	2	−0.21 (−0.44 to 0.03)	0.08
	278	3	−0.12 (−0.34 to 0.10)	0.30
	270	4	−0.12 (−0.35 to 0.12)	0.34
**Whole body BMC, g**	278	1	−224 (−301 to −147)	<0.001
	278	2	−98 (−165 to −30)	0.005
	278	3	−60 (−120 to 1)	0.05
	270	4	−69 (−138 to −1)	0.05
**Whole body less head BMC** c **, g**	278	1	−214 (−283 to −146)	<0.001
	278	2	−97 (−156 to −39)	0.001
	278	3	−61 (−112 to −10)	0.02
	270	4	−73 (−134 to −13)	0.02

The Z scores were equipment-, sex-, and age-specific. Participant's age was included in models for BMAD and BMC by a piecewise linear function allowing the effect of age to differ before and after age 20 y. aBMD denotes areal bone mineral density.

a Models: 1, sex; 2, sex and current height; 3, sex, current height, and BMI; 4, sex, current height, and exercise intensity.

b Negative values indicate lower level in VLBW individuals.

c Whole body less head BMC contains the whole body with head excluded.

### Perinatal and Neonatal Factors

Exposure to maternal smoking during pregnancy was not associated with the BMD Z scores. Because of multiple etiologies for VLBW birth, we evaluated BMD Z scores separately in three subgroups of VLBW participants: Whereas VLBW SGA participants (*n = *49) and those with exposure to premature rupture of the membranes (*n = *24) differed less from the term-born participants, this difference was larger in those with exposure to chorionamnionitis (*n = *26); in comparison to the term-born participants, they had lower Z scores: in the lumbar spine by 0.96 (0.50–1.41), in the femoral neck by 0.80 (0.38–1.22), and in the whole body by 0.56 (0.12–0.99). Gestational age at birth among this subgroup was the shortest: mean 27.9, SD 1.9 wk. Our results of lower BMD Z scores among VLBW than among term-born participants remained unchanged after reanalyzing our data to include only a portion of the VLBW participants: only singletons, only appropriate for gestational age (AGA) participants, or only those without a history of chorionamnionitis.

A longer duration of gestation was analyzed separately for those 95 participants born with VLBW but not SGA, and was associated with higher BMD Z scores: for each additional week of gestation, the values were 0.19 units higher in the lumbar spine (95% CI 0.06–0.32, *p = *0.004), 0.16 units higher in the femoral neck (0.04–0.28, *p = *0.009), and 0.13 units higher in the whole body (0.01–0.25, *p = *0.03). Data on weight Z scores were available at 36 wk postmenstrual age for 124 VLBW participants and at 40 wk for 87 VLBW participants. None of the BMD Z scores were associated with weight Z scores at birth or at 36 or at 40 wk of postmenstrual age (*p*≥0.13, linear regression), or with magnitude of Z-score change between these time points (*p*≥0.10, linear regression). Placental weight divided by birth weight was unrelated to the BMD Z scores. Further, no statistically significant correlations emerged between any of the BMD Z scores and duration of mechanical ventilation or oxygen therapy (*p*≥0.27, Spearman's correlation). Likewise, those with and those without a culture positive sepsis during neonatal intensive care had similar BMD Z scores.

### Lifestyle, Socioeconomic Status, and Medications

Adjustment for exercise intensity partially attenuated effects of VLBW (Table 4), whereas adjustment for exercise frequency or duration had little influence. Vitamin D and calcium intake data were available for 112 VLBW and 86 term-born participants. In terms of gender distribution, birth weight, and current body height, participants with food-diary data were similar to those without it. Data availability was also unrelated to study outcomes. Adjustment for calcium or vitamin D intake had no impact on differences in BMD values between VLBW and term-born participants. Similarly, adjustment for family socioeconomic status, as indicated by parental education, had no influence on these differences. Because pulmonary disorders and therapeutic glucocorticoids may affect BMD, we reanalyzed the data after simultaneous exclusion of those participants with a history of bronchopulmonary dysplasia (*n = *26, all VLBW) and those with current use of inhaled glucocorticoids (six VLBW participants and four term participants), systemic glucocorticoids (one and zero participants), or inhaled bronchodilators (14 and seven participants); the BMD results remained unchanged.

## Discussion

We show that young adults born prematurely at VLBW (<1,500 g), have, at ages 18 to 27 y, lower lumbar spine, femoral neck, and whole body BMD than do their term-born peers of normal birth weight. Differences in BMD Z scores between groups were considerable: up to 0.51 units in the lumbar spine and 0.56 units in the femoral neck. VLBW adults' smaller body size and lower exercise intensity explained only a portion of these differences; after adjusting for height, VLBW participants' lumbar spine Z score was still almost twice as likely to fall below −1.0. Lumbar spine BMD turned out to be lower also in an alternative analysis using BMAD, an index that corrects for bone size. Shorter final body height has been also previously reported in both VLBW men (3–5 cm) and women (1–8 cm) [26]–[28]. Small body size has been suggested, by some studies, to explain low bone mass in peripuberty and adolescence of those born premature [3],[4],[5],[29], whereas Wang et al. showed that VLBW individuals had reduced bone mass at ages 5 to 10 y even after adjustment for height, lean mass, or bone area [30]. Our results support the suggestion that VLBW adults attain only subnormal peak bone mass although this may partially be attributable to their small body size.

Low bone mass, microarchitectural bone deterioration, and increased fracture risk characterize osteoporosis [31], a significant public health concern in the Western world [32]. Its etiology is multifactorial, including both genes and environment [33]. Bone mass accrues with age, with at least 90% of peak bone mass acquired by age 18 y [14],[34]–[37], and with bone loss beginning at ages 35 to 45 y. Peak bone mass is regarded as the most important determinant of osteoporosis and fractures in later adulthood [38]. This underscores the importance of bone mass accrual in early childhood and during puberty: Any childhood disease process which reduces the rate or magnitude of bone mass accrual may significantly impact peak bone mass [39].

Areal BMD measured by dual-energy X-ray absorptiometry is a good surrogate marker of bone strength and presently serves to define and diagnose osteoporosis [31], with several large prospective studies documenting a strong relationship between areal BMD and probability of fracture [40],[41]. In adults, a 1-unit decrease in BMD Z score is associated with a doubling or even tripling of fracture risk [38],[40],[42]. Throughout adulthood, individuals' scores have a strong tendency to maintain the same position within the BMD distribution, showing a correlation of 0.93 between individual measurements at ages 25 and 44 y [43]. Although the prevalence of vertebral compressions was not higher in VLBW individuals in young adulthood and the lower BMD was partially explained by a smaller body size, the observed lower bone mass may translate into increased susceptibility to fracture and skeletal morbidity during later life.

The low vitamin D intake reported by both VLBW adults and those born at term, in a representative subgroup with food-diary data, is in accord with other Finnish findings on young adults [44]. The low intake may explain the slightly negative BMD Z scores in even our term-born group. Vertebral compression frequencies, however, were in line with those reported in a Danish study of young men [45]. Our observations underscore the importance of a comparison group from the same population. The differences in BMD between our two groups were not due to vitamin D or calcium intake, and low leisure-time exercise intensity reported by the VLBW participants explained the differences only partially. However, nutritional counseling and especially promotion of weight-bearing activity in former VLBW infants are essential for osteoporosis prevention.

We detected no effects of slow intrauterine growth, whereas a short duration of gestation was associated with unfavorable skeletal health in adulthood, in accordance with findings among prepubertal boys [30]. Another study among young adults with VLBW showed no gestational-age effects on skeletal health, but included only 25 participants [3]. Low BMD Z scores were associated with chorionamnionitis but not with relative placental weight. These post hoc observations need to be confirmed but they may suggest that several perinatal factors, including but not limited to immaturity and infection, may partially determine future bone health.

### Study Limitations

Although our original cohort comprised the entire population of VLBW infants discharged alive after neonatal intensive care in the Helsinki area, our study participants may not be representative of our original cohort. Our participation rate was, however, similar to or higher than that in most clinical follow-up studies in adults born preterm [46]–[48]. Moreover, our results are based on internal comparisons within the study sample; nonparticipation would introduce bias only if the effect of VLBW on adult outcomes differed among nonparticipants. After our participants were born, improvements in perinatal care have changed the characteristics of VLBW survivors. Recently born VLBW survivors, however, also have lower-than-expected bone mass in infancy and childhood [49], which might justify generalizing our results and indicate that today's survivors will show impaired skeletal health in adulthood. Neurosensory impairment, a possible cause of nonparticipation of those individuals with the greatest likelihood of impaired skeletal health, may have caused underestimation of bone mineral deficit in VLBW individuals. We were able to study BMD only once and thus unable to exclude an alternative explanation that our findings could be explained by a delayed tempo of bone mineralization rather than lower peak bone mass. However, we are unaware of any data suggesting delayed skeletal maturation after preterm birth, rather related conditions such as low birth weight and intrauterine growth retardation are associated with rapid maturation [50]. We used a 3-d food diary to estimate vitamin D intake. This method has limitations and studies with more precise methods and longitudinal follow-up are needed to evaluate the role of vitamin D in bone mass accrual in VLBW individuals. Finally, no history of osteoporotic fractures was available for our study participants; it will obviously be uninformative to assess the clinical significance of the reduced BMD in the light of life-time fracture risk before these participants grow older.

### Conclusion

We conclude that healthy young adults born preterm with VLBW, as compared with their term-born counterparts, show signs of significantly compromised skeletal health in adulthood, including a 2-fold risk for having a lumbar spine BMD Z score below −1.0. This finding may predict symptomatic osteoporosis and increased fracture rates. Promotion of adequate nutrition with sufficient calcium and vitamin D, and an increase in weight-bearing exercise are thus important for former VLBW infants at all ages.

## Abbreviations

aBMD, areal bone mineral density; BMAD: bone mineral apparent density
BMC: bone mineral content
BMD: bone mineral density
BMI: body mass index
CI: confidence interval
IQR: interquartile range
SGA: small for gestational age (birth weight SD score≤−2.0)
SD: standard deviation
VLBW: very low birth weight (<1,500 g)
